# Hierarchical One-Dimensional Ammonium Nickel Phosphate Microrods for High-Performance Pseudocapacitors

**DOI:** 10.1038/srep17629

**Published:** 2015-12-03

**Authors:** Kumar Raju, Kenneth I. Ozoemena

**Affiliations:** 1 Energy Materials Unit, Materials Science and Manufacturing, Council for Scientific & Industrial Research (CSIR), Pretoria 0001, South Africa; 2 School of Chemistry, University of the Witwatersrand, Johannesburg 2050, South Africa

## Abstract

High-performance electrochemical capacitors will drive the next-generation portable, flexible and wearable electronics. Unlike the conventional all-carbon supercapacitors (electric double layer capacitors, EDLC) with high power but poor energy density, pseudocapacitors capitalize the high energy density inherent to reversible redox reactions and provide a facile means to enhancing the energy ratings of supercapacitors. The high length-to-diameter ratio and anisotropic character of 1-D architecture makes them suitable for use in energy storage. For the first time, we report 1-D microrod structures (~ 36 nm width) of ammonium nickel phosphate hydrate (ANP_mr_) as a pseudocapacitor with high energy rating and power handling. To confirm the data, the ANP_mr_-based pseudocapacitor was subjected to various configurations (i.e., half-cell, symmetric, asymmetric, and flexible all-solid-state) and in each case it gave excellent values compared to any accessible literature to date. We clearly demonstrate that a flexible all-solid-state ANP_mr_-based pseudocapacitor achieved high areal capacitance of 66 mF cm^−2^ with extra-ordinary energy (21.2 mWh cm^−2^) and power (12.7 mW cm^−2^) densities. This work opens doors for a facile, robust and scalable preparation strategy for low-cost, earth-abundant electrode materials for high-performance pseudocapacitors.

A considerable amount of research attention has continued to be devoted to renewable and clean energy technologies globally. The research on improving the electrical energy storage is crucial for increasing the supply of more energy from renewable sources to curb the present energy challenges^1^,^2^,^3^,^4^,^5^. There are two main classes of electrochemical capacitors (ECs); the all-carbon based electrical double layer capacitors (EDLCs) that store energy by charge-separation at the solid-electrolyte interface, and the pseudocapacitors that store energy by redox or Faradaic processes. EDLCs (also known as the supercapacitors or ultracapacitors) are important energy storage devices with adequate energy and high power densities compared to conventional electrochemical energy storage systems such as batteries and fuel cells^6^,^7^. An extraordinary storing of electrical energy with exceptional power has been projected to increase the awareness and development of important technologies such as hybrid electric vehicles, portable electronics and power-saving units. It is well-established that the performance of supercapacitor-driven technologies is dependent on the physicochemical properties of their electrode materials^8^. Flexible and wearable electronics have begun to attract intense research interests due to their reliability, ease of handling, and the great promises to be used as flexible energy storage devices^9^. Pseudocapacitors have emerged as the most successful and substantial electrical energy storage devices for the flexible and wearable electronics. However, one of the major short-comings of the pseudocapacitor over their supercapacitor counterpart is their poor rate-capability compared to supercapacitor^10^. Therefore, the need for the development of high-performance pseudocapacitor materials is urgent.

Hierarchical 1-D and 2-D materials maximize the supercapacitive properties due to their unique ability to permit ion propagations^11^,^12^. Phosphate-rich materials (PRMs), such as mesoporous NH_4_NiPO_4_.H_2_O nanoalmonds^13^, 1-D layered NH_4_CoPO_4_.H_2_O microrods^14^ and 2-D VOPO_4_ nanosheets^15^ have been reported as high-performance pseudocapacitors. For example, the energy and power densities of these PRMs range between 30–140 Wh kg^−1^ and 1–27 kW kg^−1^ for 3-electrode configurations. One of the major challenges with the PRMs is their low electrical conductivity, which explains why, for example, the VOPO_4_ nanosheets had to be integrated with high-electrical-conducting graphene sheets^15^. There is a need therefore to make PRMs that allow for compatible conduction pathways in their structures for improved redox-activity and pseudocapacitive behaviour. 1-D materials have been known to greatly influence space-confined transport phenomena thereby improving the charge accumulation and redox reactions^11^,^16^. Indeed, the major attractions of 1-D architecture include their high length-to-diameter ratio and anisotropic character. The small diameter of the 1-D architecture is important as it allows for enhanced accommodation of possible large volume changes, thus preventing possible cracking or fracturing of the structures usually observed in bulk or micron-sized materials. It is possible for ions and electrons to be simultaneously integrated into 1-D architectures, thereby making them ionically and electronically conductive. Importantly, 1-D architecture are characterised by large surface-to-volume ratio that permit efficient contact between the active mass of the electrode and the electrolyte, promoting high-rate capability. It is without doubt therefore that an important strategy for achieving high-performance PRMs is the preparation of their hierarchical 1-D architectures such as the wire-like or rod-like morphology. To our knowledge, rod-like PRMs do not exist.

Motivated by the above advantages of 1-D materials in pseudocapacitor applications, and the current challenges identified in the literature, this work describes the first synthesis of 1-D NH_4_NiPO_4_ microrods (with nanometric width) with a view to increasing the energy and power densities. Herein we introduce a facile strategy to synthesize various morphologies of NH_4_NiPO_4_.H_2_O by hydro/solvothermal route in ethylene glycol (EG), water and mixed solvents of EG/H_2_O using nickel-based acetate and ammonium phosphate without the use of a template and additives. We show that when subjected to various experimental conditions from half-cell and symmetric to asymmetric and flexible all-solid-state configurations, the NH_4_NiPO_4_ microrods still maintained excellent performance.

## Results and Discussion

**Figure 1:**
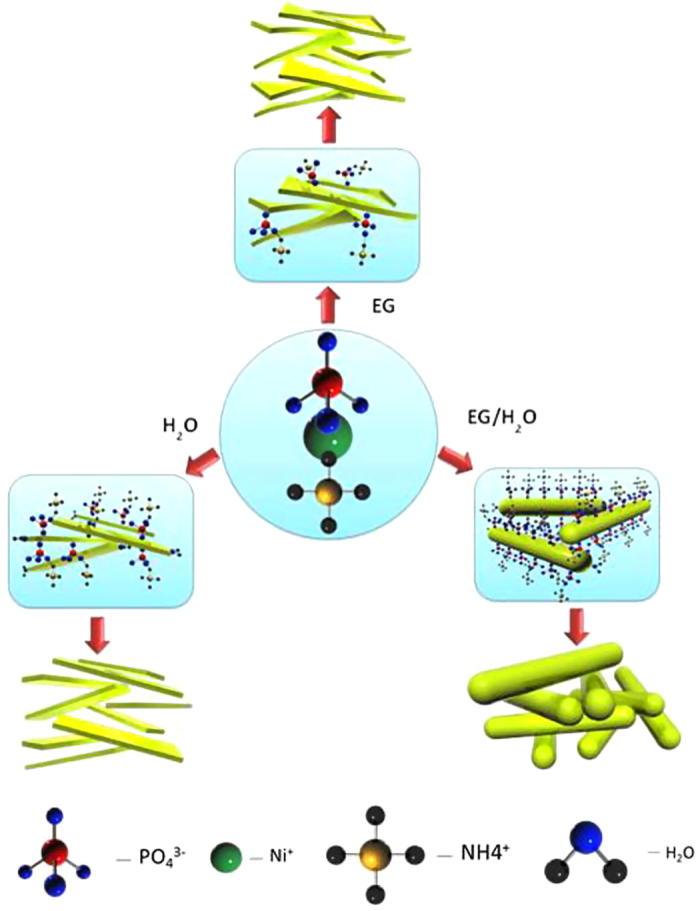
(Ozoemena): Schematic representation of various formations of ANP materials.

**Figure 2:**
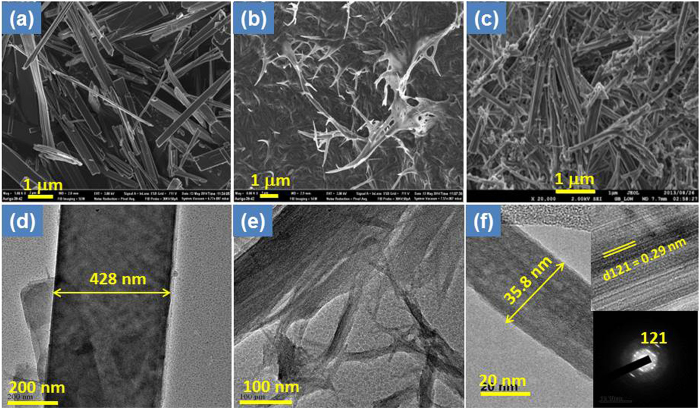
(Ozoemena): SEM micrograph of as-synthesized various morphologies of (**a**) ANP_mp_ (microplatelets), (**b**) ANP_md_ (microdendrites) and (**c**) ANP_mr_ (microrods), respectively. TEM and HR-TEM images (**d**) ANP_mp_ with diameter of 428 nm, (**e**) ANP_md_ microdendrites with >100 nm diameter and (**f**) single nanorod of ANP_mr_ with 35.8 nm diameter, the inset of 2f showing lattice fringes corresponding to the (121) plane of microrods and their SAED pattern.

A schematic representation of the synthetic strategy adopted for the formation of three different nanostructures of NH_4_NiPO_4_.H_2_O is summarised in Fig. 1. The microdendrites of NH_4_NiPO_4_.H_2_O (ANP_md_), microplatelets (ANP_mp_) and microrods (ANP_mr_) were synthesized in EG, water, and EG/water mixture via facile solvo/hydrothermal processes, respectively. The morphology and size of as-synthesized NH_4_NiPO_4_.H_2_O were confirmed with the SEM and TEM analyses. Figure 2 (a–f) shows the SEM and TEM images of ANP_md_, ANP_mp_ and ANP_mr_ showing the unique formation of different morphologies of microplatelets, microdendrites and microrods. It is interesting to note from Fig. 2 that the obtained microplatelets (Fig. 2a) have average dimensions of 400-600 nm in length and nearly 428 nm in diameter (Fig. 2d). Figure 2b,e illustrate the SEM and TEM images of ANP_md_ with average dimension of 100–300 nm in length. As shown in Fig. 2c, the obtained microrods have average dimensions of 200–300 nm in length and 35–40 nm in diameter, with few of them as nanorods. As illustrated by the HRTEM image, (Fig. 2f and insets), the observed microrods (with nanometric width) are polycrystalline in nature with clear lattice fringes. The d-spacings of the lattice fringes is found to be 0.29 nm, corresponding to the (121) plane of ANP_mr_ along with SAED pattern corresponding to (121) plane. Note that these microrods could only be formed at 48 h as shown from the SEM and TEM images (Supplementary Information, Figure-S1).

From the results, it is evident that spatial localization of water molecules in solvent mixture is critical in mediating the shape growth; the highly viscous solvent EG (*η* = 21 mPa s, 20°C) restrains the mobility of reactants compared to water (*η* = 1.0087 × 10^−3^ mPa s, 20 °C). The solubility and mobility of reactants considerably favours the homogeneous nucleation process when an appropriate amount of EG/H_2_O is used^17^. In general, the formation mechanism of the microstructures seems to occur via the hydrolysis of acetate to acetic acid and hydroxide ion in aqueous solution, followed by the reaction between the PO_4_^3−^, NH_4_^+^ and Ni^2+^ ions. The diffusion of the active sites of PO_4_^3−^, NH_4_^+^ and Ni^2+^ ions increases with a rise in temperature thus enhancing the nucleation process. The viscosity of EG decreased with increase in temperature thereby facilitating fast nucleation by reducing the interlayer spacing thus enhance the anisotropic growth of nanorods^18^. The NH_4_NiPO_4_.H_2_O layers are formed by sharing the highly distorted NiO_6_ octahedra corners with cross-linked distorted PO_4_^3−^ tetrahedra and NH_4_^+^ ions inserted between the inorganic layers via hydrogen bonding^19^. There are other 1-D materials, such as LnPO_4_ and CePO_4_, whose formation is driven by diffusion-controlled growth mechanism, i.e., attachment of infinite linear chains along the axis of its crystalline phase^20^. The structural arrangement of NH_4_NiPO_4_.H_2_O also contains open channels of octahedra along the parallel and perpendicular axis in (010) and (001) planes which may tend to form linear chain extending of octahedral along the axis. Additionally, a drastic change in morphology has also been reported when using different reactants. For instance, Zhao *et al.*^13^ recently reported that nickel nitrate gives almond-like NH_4_NiPO_4_.H_2_O whereas nickel acetate gives dendrite-like NH_4_NiPO_4_.H_2_O morphology. The nitrate anion, which is a weak base and a good-leaving group, can easily substitute with one another and proceeds to 3-D growth process, whereas strong base of acetate anions surrounding the cation impede the 3-D growth. However, acetate ions and equal mixture of water and EG play a crucial role in the formation of unique morphologies of 1-D NH_4_NiPO_4_.H_2_O materials, a facile and scalable approach which is more suitable to be extended to the preparation of various 1-D and 2-D ammonium metal phosphate (NH_4_MPO_4_.H_2_O, where M = Ni, Mn, Fe, Co, etc) materials.

**Figure 3:**
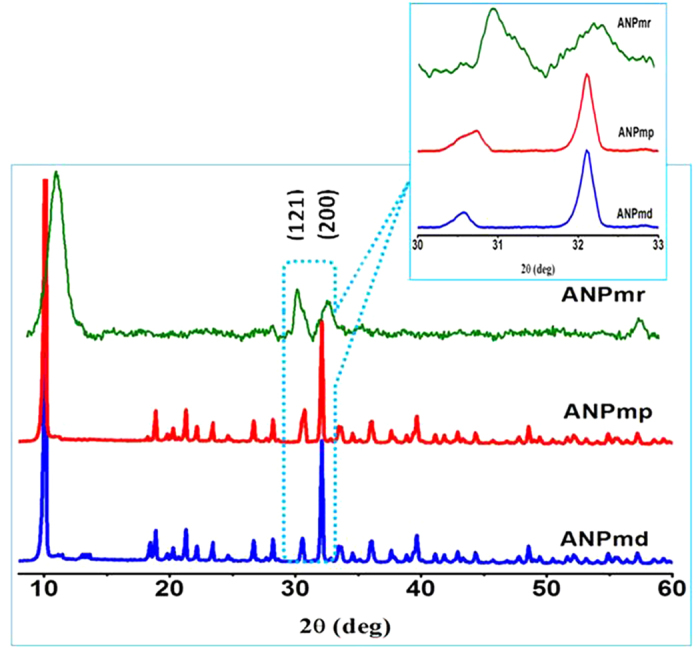
(Ozoemena): XRD pattern of ANP samples of ANP_mp_, ANP_md_ and ANP_mr_. The inset is an expanded view of (121) and (200) peaks.

Figure 3 shows the XRD pattern of NH_4_NiPO_4_.H_2_O samples of ANP_mp_, ANP_md_ and ANP_mr_. The XRD patterns indicate the characteristic peaks of a pure phase of NH_4_NiPO_4_.H_2_O and all the observed peaks can be readily indexed to a pure orthorhombic phase (space group: Pmn2) with the cell parameters of *a* = 5.425 Å, *b* = 8.77 Å and *c* = 4.31 Å in accordance with the JCPDS card no 86-0585. Interestingly, an increase in intensity of the (121) peak is observed compared to the (200) peak infers the preferential orientation or anisotropic growth along the c-axis (insert Fig. 3). The broad peak of the ANPmr is a clear indication that its particle size is smaller than those of the ANP_mp_ and ANP_md_.

### Electrochemical characterization of three-electrode system

**Figure 4:**
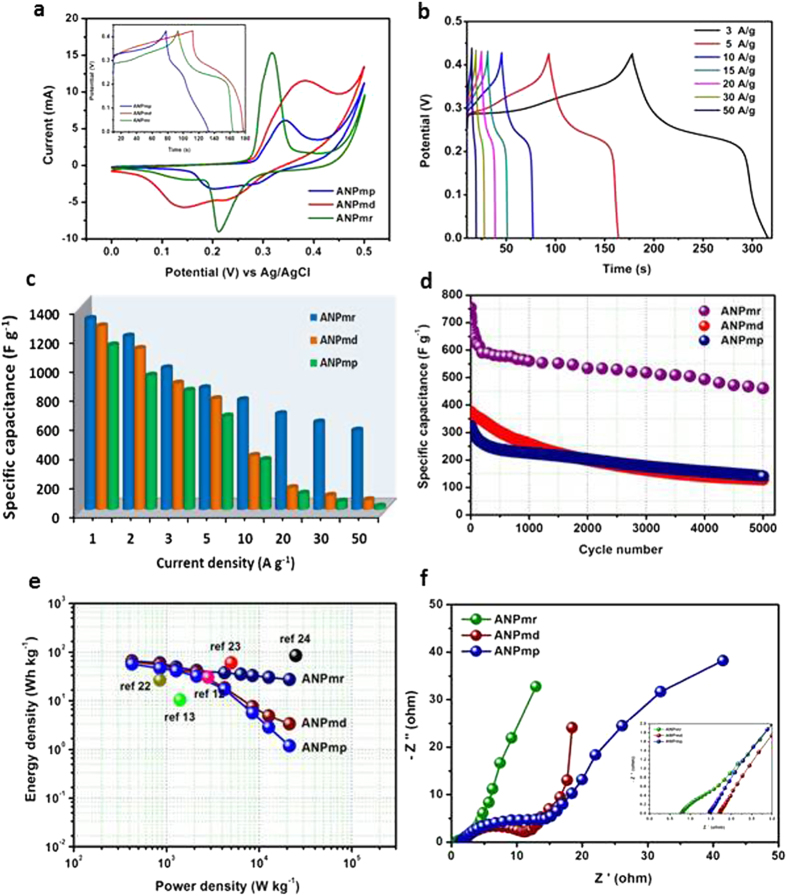
(Ozoemena): Comparative supercapacitive performance of the three different ANP electrodes using *three-electrode* (half-cell) configurations with nickel foam as the working electrode and 3M KOH as the aqueous electrolyte: (**a**) Typical CV curves at 10 mVs^−1^, insert shows the gavanostatic charge-discharge (GCD) curves at 5 A g^−1^; (**b**) GCD curves between 3 and 50 A g^−1^; (**c**) Specific capacitance vs. current density, i.e., rate capability test; (**d**) durability test for 5,000 continuous GCD cycles at 10 A g^−1^; (**e**) Ragone plots compared with other similar electrode materials reported in literature using 3-electrode configurations^12^,^13^,^22^,^23^,^24^; (**f**) Nyquist plot of 3 M KOH solution (inset; magnified view).

Figure 4 compares the electrochemical performance of the three-electrode configurations of the three microstructures. The cyclic voltammetric evolutions (Fig. 3a) depict a redox couple arising from the redox-active nickel (Ni^2+^/Ni^3+^), confirming the pseudocapacitive behaviour of the NH_4_NiPO_4_.H_2_O. The emergence of the redox couple is represented as follows:^14^,^21^


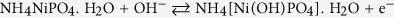


The microrods showed well-defined electrochemistry with large current density and narrower peak-to-peak potential (Δ*E*_p_ ≈ 150 mV at 20 mVs^−1^) compared to the >200 mV shown by the microdendrites and microplatelets, meaning that ANP_mr_ exhibits better electrochemical reversibility and faster electron transfer kinetics. Also, unlike the others, the microrods showed no additional oxidation peaks, indicating that the only oxidation process is that of Ni^2+^/Ni^3+^ without any other phase changes.

Figure 4b shows typical charge/discharge curves of the ANP_mr_ at different current densities (1 to 50 A g^−1^). At all the current densities investigated (Fig. 4c), the ANP_mr_ electrode showed the best performance compared to the ANP_md_ and ANP_mp_, achieving remarkable a maximum reversible specific capacity of ~1400 F g^−1^. Interestingly, the ANP_mr_ showed an extra-ordinary high rate capability proven by the high capacitance of 545 F g^−1^ at a very high current density of 10 A g^−1^ which is extremely high in comparison with the capacitance value reported in the literature to date. The high value reflects the effective ion migration even at a higher speed which is influenced by high surface area of microrods with minimum diffusion length of ion accessibility. Upon continuous cycling, the electrode experienced capacitance loss at the initial cycles, stabilized at about 200 cycles and then retained nearly ~80% of its original specific capacitance after 5,000 cycles (Fig. 4d). Figure 4e highlights the Ragone plot of the calculated power and energy densities. The results are higher than most recently reported supercapacitor nanomaterials based on 3-electrode configurations (Supplementary Information, Table-S1).

To provide further insights into the pseudocapacitive behaviour of the ANP materials, EIS experiments were conducted at open-circuit voltage at room temperature. Figure 4f compares the EIS (Nyquist plots) of the three ANP-based electrodes. The EIS data were satisfactorily fitted with the electrical equivalent circuit (EEC) comprising two Voigt RC elements, involving a series resistance (*R*_s_), charge-transfer resistance (*R*_ct_) and constant-phase elements (CPE or Q). As summarised in the Table-S2 (Supplementary Information) the *R*_s_ of the ANP_mr_ (*ca.* 0.8 Ω) is smaller than those of the ANP_mp_ (1.48 Ω) and ANP_md_ (1.72 Ω). Also, the total *R*_ct_ values of the ANP_mr_ (*ca.* 25.6 Ω) is smaller than those of the ANP_md_ (*ca.* 31.4 Ω) and ANP_mp_ (*ca.* 41.4 Ω). These results clearly indicate that the rod-like morphology provides the least internal resistance of the electrode and permits faster charge transportation compared to other morphologies investigated in this study. In addition, from the Fig. 4f, the ANP_mr_ showed near-vertical line as expected of a high-performing pseudocapacitance compared to others. The “knee” or “onset” frequency (*f*_o_), which is a measure of the power capability of a supercapacitor, decreases as ANP_mr_ (5 kHz) > ANP_md_ (1.7 kHz) > ANP_mp_ (1.18 kHz), confirming the higher energy-storage capability of the ANP_mr_ over other electrodes. The experimentally observed impedance curve was best fitted with the equivalent circuit and the calculated value of ESR of ANP_mr_, ANP_md_ and ANP_mp_ were found to be 0.79, 1.72 and 1.48 Ω. It is noted that the impedance of CPE is defined as equation (1):


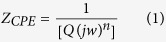


where *Q* represents the frequency-independent constant relating to the surface electroactive properties, *w* is the radial frequency, the exponent *n* arises from the slope of log Z vs. log f (and has values −1 ≤ *n* ≤ 1). If n = 0, the CPE behaves like a pure resistor; *n* = 1, CPE behaves as a pure capacitor, n = –1, CPE behaves as an inductor; while *n* = 0.5 corresponds to Warburg impedance (*Z*_w_) which is related to the diffusion of the ions. The *n* values observed for the ANP_mr_ (*n*_1_ = 0.58 and *n*_2_ = 0.53) while other electrodes were *ca.* 0.8, which suggests that the rod-like structure allows for improved ion diffusion than others. Further, the impedance curves were measured after 5,000 consecutive charge–discharge cycles (Supplementary Information, Figure-S2).

### Symmetric pseudocapacitors in alkaline electrolyte (3 M KOH)

**Figure 5:**
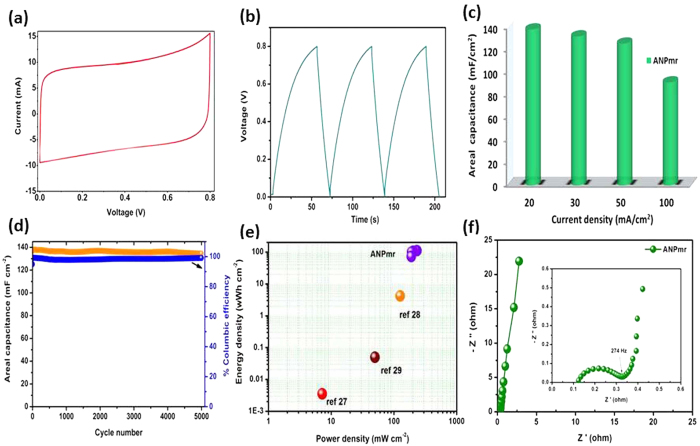
(Ozoemena): Electrochemical performances of symmetric pseudocapacitors of ANP_mr_ coated on carbon cloth in 3M KOH: (**a**) Typical cyclic voltammogram obtained at a scan rate of 10 mVs^−1^; (**b**) galvanostatic CD profiles of ANPweg at the current density of 10 mA cm^−2^; (**c**) Areal capacitance calculated from CD curves as a function of current density; (**d**) Cycling stability and coulombic efficiency from 5,000 continuous charge-discharge cycles at 10 mA cm^−2^; (**e**) Ragone plot of A ANP_mr_ symmetric supercapacitor compared with other symmetric supercapacitor values reported in literature^27^,^28^,^29^; and (**f**) Nyquist plot of ANP_mr_ symmetric supercapacitor (inset; magnified view).

Considering the high-performance of the ANP_mr_ electrode at half-cell configuration, subsequent studies on full-cell pseudocapacitor devices were devoted to the ANP_mr_. Figure 5 summarises the performance of the ANP_mr_ as a symmetric pseudocapacitor in an aqueous alkaline electrolyte (3 M KOH) using carbon cloth as substrate/current collector, showing typical CV curve at a scan rate of 10 mVs^−1^ (Fig. 5a), charge-discharge curves at current density 10 mA cm^−2^ (Fig. 5b), areal capacitance (Fig. 5c), cycle stability (Fig. 5d), Ragone plots (Fig. 5e) and Nyquist plot (Fig. 5f) of ANP_mr_ symmetric pseudocapacitors. The symmetric pseudocapacitor showed satisfactory rate capability, evident at different scan rate and current densities (Supplementary Information, Figure-S3). Interestingly, the electrode gave high areal capacitance of 138 mF cm^−2^ at 20 mA cm^−2^, 92% of which (126 mF cm^−2^) was retained even at a current density of 50 mA cm^−2^. The high rate capability can be attributed to the microrods maintaining their excellent structural stability and charge propagation even at higher current densities. It was found that the ANP_mr_ has the highest areal capacitance compared with recently reported symmetric supercapacitor materials (*cf.* Supplementary Information, Table-S3). More importantly, the areal capacitance retain more than 97% of its initial values after 5000 continuous charge–discharge cycles with 100% columbic efficiency (Fig. 5d). As shown in Fig. 5e, the ANP_mr_ delivered the highest energy and power densities of 69 mWh cm^−2^ and 145 mW cm^−2^ at a current density of 20 mA cm^−2^ and was found that the ANP_mr_ has energy values compared to that of literature values. As shown in the EIS spectra (Fig. 5f), the observed high frequency intercept show that ANP_mr_ (0.13 Ω) have much smaller ESR with inclined vertical line after the semicircle with the response time of 8 ms was lower than the values reported in liquid electrolyte used supercapacitors, onion- like carbon (26 ms)^25^ and biscrolled yarn (17 ms)^26^. As summarised in the Table-S2 (Supplementary Information), the ANP_mr_-based symmetric cell gave very small *R*_s_ (*ca.* 0.13 Ω) and total *R*_ct_ (*R*_ct1_ + *R*_ct2_ *≈* 33 Ω), with each *n* value (*n*_1_ or *n*_2_) greater than 0.8 but less than 1, clearly confirming the pseudocapacitive properties of the ANP_mr_ when deployed in full-cell system.

### Asymmetric pseudocapacitors in neutral aqueous electrolyte (1 M Na_2_SO_4_)

**Figure 6:**
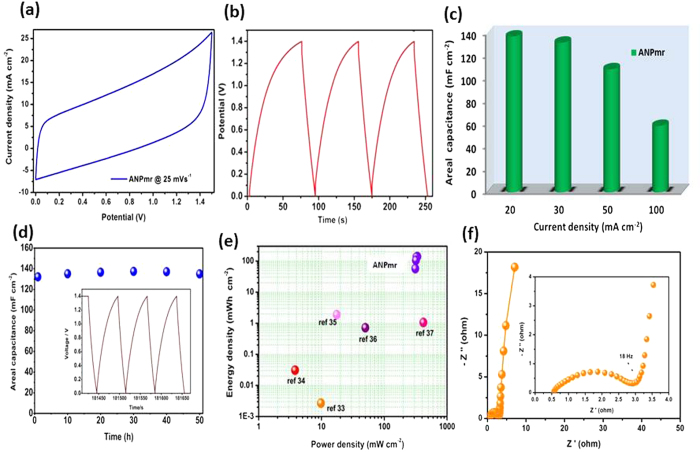
(Ozoemena): Electrochemical performances of asymmetric pseudocapacitors of ANP_mr_//AC coated on carbon cloth 1M Na_2_SO_4_: (**a**) Typical specific capacitance calculated from CV curve against voltage at a scan rate of 25 mVs^−1^; (**b**) galvanostatic CD profiles at 10 mA cm^−2^; (**c**) Areal capacitance from CD curves as a function of current density; (**d**) Typical voltage-holding (floating) curves acquired for 50 h at the voltage of 1.4 V and at 10 mA cm^−2^, inset is an example of CD curves after 50 h voltage-holding; (**e**) Ragone plot for this work and similar symmetric supercapacitors reported in literature^33^,^34^,^35^,^36^,^37^; (**f**) Nyquist plot of ANP_mr_//AC (inset; magnified view).

We also prepared asymmetric pseudocapacitors in order to further increase the energy density of the device. Typically, ANP_mr_ coated carbon cloth electrodes were used as positive and activated carbon (Norit^®^ supra) coated carbon cloth electrode as negative in 1M Na_2_SO_4_ neutral aqueous electrolyte. The cyclic voltammograms of ANP_mr_ (Fig. 6a) obtained at a scan rate of 25 mVs^−1^ shows rectangular shapes. Figure 6b show the charge-discharge at a current density of 10 mA cm^−2^, the cell gave high areal capacitance of 221 mF cm^−2^ at 20 mA cm^−2^, 90% of (201 mF cm^−2^) which was retained even at a current density of 50 mA cm^−2^. It was found that this asymmetric capacitor has best areal capacitances compared to many nanostructured electrodes reported earlier in asymmetric capacitors, such as H-TiO_2_ @ MnO_2_ (0.9 F cm^−3^)^30^, TiO_2_/NiO nanotube array (2.9 mF cm^−2^ at 0.4 mA cm^−2^)^31^ and Fe_3_O_4_-SnO_2_ core-shell nanorod film (7 mF cm^−2^)^32^.

As shown in Fig. 6d, 50 h voltage-floating tests show excellent capacity retention for the ANP_mr_ cell with areal capacitance of 135 mF cm^−2^, and long cycle stability with an almost 100% columbic efficiency. Ragone plot of ANP_mr_ asymmetric capacitors (Fig. 6e) exhibits extraordinary energy (134.6 mWh cm^−2^) and power (325.6 mW cm^−2^) densities at a current density of 20 mA cm^−2^. These values are much higher when compared with other asymmetric pseudocapacitors (Supplementary Table-S3). Figure 6f shows the Nyquist plot of ANP_mr_//AC asymmetric pseudocapacitor in 1M Na_2_SO_4_ neutral aqueous electrolyte showed very small ESR (0.55 Ω) with inclined vertical line with the response time of 18 s. From Table-S2 (Supplementary Information), the ANP_mr_-based asymmetric cell showed similar EIS behaviour as its symmetric cell counterpart; very small *R*_s_ (*ca.* 0.55 Ω) and total *R*_ct_ (*ca.* 41 Ω), with each *n* value (*n*_1_ or *n*_2_) greater than 0.8 but less than 1, further corroborating that the pseudocapacitive behaviour of the ANP_mr_ when used in full-cell system. In addition, it may be observed that the CV curve (Fig. 5a) is higher in the positive direction than in the negative side. This behaviour is related to the series resistance of the asymmetric configuration; the *R*_s_ value of the asymmetric configuration is much higher (*ca.* 0.55 Ω) than the symmetric counterpart (*ca.* 0.13 Ω), which explains why we did not observe this for the symmetric configuration.

### All-solid-state flexible symmetric pseudocapacitors

**Figure 7:**
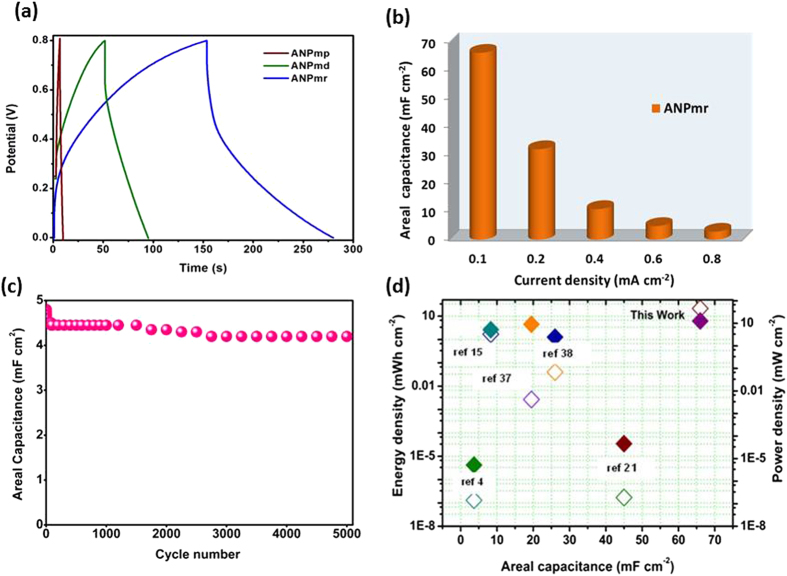
(Ozoemena): Electrochemical performances of all solid-state flexible symmetric pseudocapacitors fabricated on a carbon cloth with PVA/KOH polymer electrolyte: (**a**) Typical galvanostatic CD profiles at 0.2 mA cm^−2^; (**b**) Areal capacitance from CD curves as a function of current density; (**c**) durability test at 0.6 mA cm^−2^; and (**d**) Ragone plot of ANP_mr_-based all-solid-state flexible symmetric pseudocapacitors compared with similar systems reported in the literature^4^,^15^,^21^,^37^,^38^.

Finally, we explored the performance of the as-prepared ANP materials (ANP_mp_, ANP_md_ and ANP_mr_) as all-solid-state flexible symmetric pseudocapacitors using PVA-KOH polymer electrolyte and carbon cloth as substrate/current collector (Fig. 7). Also, nickel foam was used as the substrate/current collector for the ANP_mr_-based all-solid-state pseudocapacitors (see Supplementary Information, Figure-S4) but we found carbon cloth to be easier to handle, more flexible, with more improved electrochemical properties than nickel foam. Thus, all further studies were devoted to the use of carbon cloth. From every analysis, the ANP_mr_ showed better electrochemical performance than the other two ANP materials (Supplementary Information, Figure-S5). Figure 7a exemplifies typical galvanostatic charge–discharge experiments, while Fig. 6b compares the specific capacitance values obtained at various current densities (0.1–0.8 mAcm^−2^). The ANP_mr_ gave an excellent specific areal capacitance of 66 mF cm^−2^ at 0.1 mA cm^−2^ (Fig. 7b), and even at a higher current density of 0.8 mA cm^−2^ the capacitance remained as high as 3 mF cm^−2^. This value is much higher compared to the values reported for other all-solid-state symmetric and asymmetric supercapacitors (Supplementary Information, Table-S4). For example, areal capacitance achieved with ANP_mr_ was better than the electrochemical double layer microcapacitors which delivered 0.4–2 mF cm^−2^ at scan rates of 1–100 mVs^−1^ 27, and graphene or carbon nanotube based flexible supercapacitors that showed 3–50 mF cm^−2^ 28. From the prolonged cycle stability performed at the scan rate of 0.6 mA cm^−2^, the ANP_mr_ retained ~97% of its initial capacitance even after 5000 consecutive cycles (Fig. 7c). The Ragone plot (Fig. 7d) showed significantly higher energy (21.2 mWh cm^−2^) and power (12.7 mW cm^−2^) densities compared to the values reported in the literature to date for all-solid-state SCs (Fig. 6d, and Supplementary Information, Table-S4). From the Nyquist plots of ANP_mr_ (Supplementary Information, Figure-S5(e)) the equivalent series resistance (ESR) values obtained before and after 5000 cycles were 4.5 and 23 Ω, respectively. The response time (60 ms) was lower than the reported values for solid electrolytes (80 ms), and activated carbon (700 ms)^22^. Indeed, detailed examination of the EIS data of the various ANP materials in Table-S2 (i.e., ANP_mp_, ANP_md_ and the three ANP_mr_ obtained at 24, 36 and 48 h) showed an interesting trend on the conductivity of the various morphologies. The three rod-like morphologies gave the least series resistance (i.e., *R*_s_ ≈ 5 Ω) compared to the platelet-like (*R*_s_ ≈ 11 Ω) and dendrite-like (*R*_s_ ≈ 25 Ω) morphologies. The total *R*_ct_ values decrease as follows: ANP_mr@36h_ (*ca.* 140 Ω) > ANP_md_ (*ca.* 115 Ω) > ANP_mp_ (*ca.* 93 Ω) > ANP_mr@24h_ (*ca.* 32 Ω) > ANP_mr@48h_ (*ca.* 24 Ω). This is an interesting result as it clearly corroborates other electrochemical data that shows that our rod-like morphology, obtained at optimized 48 h and 200 °C, gave the best conditions for ion mobility in pseudocapacitor devices.

To further understand the reason for the high-performance of the ANP_mr_, we examined its specific surface area and porosity by performing the Brunauer–Emmett–Teller (BET) measurements (Supplementary Information, Fig. S6 showing the N_2_ adsorption–desorption isotherm of the nanorods). The observed BET surface area was 214 m^2^ g^−1^ with an average pore size distribution of 2–20 nm with a pore volume of 0.7 m^3^g^−1^ (BJH desorption), inferring the co-existence of mesoporous and microporous microrods.

**Figure 8:**
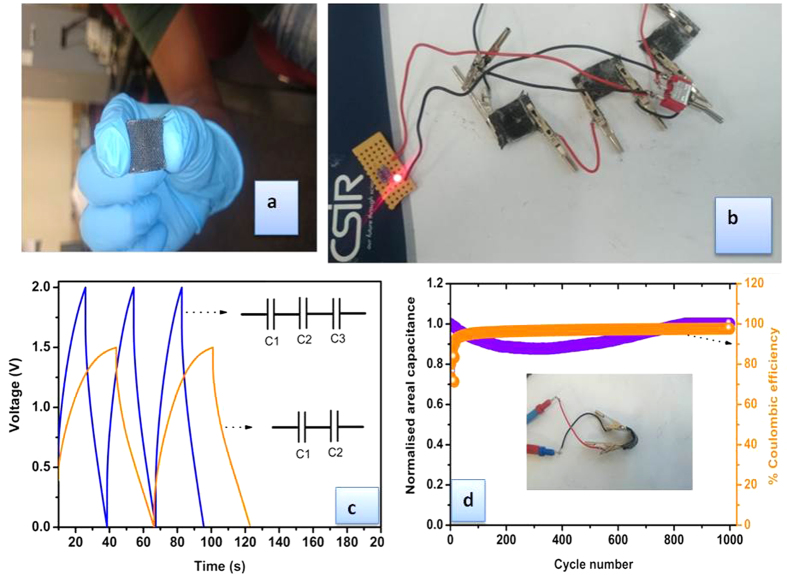
(Ozoemena): (**a**) Photograph of the as-prepared ANP_mr_-based flexible all-solid-state symmetric pseudocapacitor (ASSSP); (**b**) Typical 3 ASSSPs connected in series and lighting up a 1.67 V LED; (**c**) Charge–discharge profiles of 2 and 3 ASSSPs connected in series giving 1.5 and 2 V, respectively; and (**d**) cycle stability measured nearly at 120° bending angle and their coulombic efficiency, insert shows picture of an ASSSP bent at 120°.

As a proof of concept, Fig. 8 describes the bendability of the ANP_mr_-based all-solid-state symmetric pseudocapacitor (ASSSP) and its ability to light up a 1.67 V LED when connected in series. Interestingly, when the ASSSP was bent to nearly 120° and subjected to 1000 charge-discharge cycles, it was able to maintain its performance with *ca.* 100% coulombic efficiency.

## Conclusions

Novel NH_4_NiPO_4_.H_2_O with unique morphologies (microdendrites, microplatelets and microrods) of different supercapacitive properties has been reported. The microrod morphology gave an extraordinarily high specific capacitance, power and energy densities in half-cell and full-cell configurations (i.e., symmetric and asymmetric cells, including all-solid-state flexible pseudocapacitors) in different electrolytes. The well-aligned microrods (*ca.* 35.8 nm diameter) with meso- and microporous surface enhance ion propagation and interfacial interactions compared to the long range plates (428 nm in diameter) with larger thickness or the microdendrites with smaller branched structure (*ca.* 100 nm) with uneven surface. The all-solid-state symmetric pseudocapacitor fabricated from the ANP microrods proved it can generate power even when bent to 120° and can drive an LED when connected in series. The study proves that rod-like morphology (with ~ 36 nm width) provides a significant and promising direction for novel 1-D and 2-D materials to obtain high-performance pseudocapacitors, especially for flexible and wearable electronics. The study has opened new doors of research opportunities for this type of materials. Such opportunities include examination of other redox-active metals other than nickel, and interrogation of the impact of tuning the reported synthesis protocols (e.g., in terms of changes in temperature, solvents, reaction times, work-up conditions, etc) or new synthesis procedure on the final structures and physicochemical properties. These constitute the directions of the on-going investigations in our laboratory.

## Methods

### Synthesis and characterisation of NH_4_NiPO_4_.H_2_O nanorods (with micrometric length)

Analytical grade chemicals, Nickel (II) acetate tetrahydrate (Ni(CH_3_COO)_2_.4H_2_O), ammonium dihydrophosphate (NH_4_H_2_PO_4_) and ethylene glycol (EG) were procured from Sigm-Aldrich, and used as received without further purification. In a typical synthesis, 0.5 g of Ni(CH_3_COO)_2_.4H_2_O and NH_4_H_2_PO_4_ were dissolved thoroughly in 40 ml deionized water. Subsequently, an equal amount of EG was added into the above solution (water and EG volume ratio is 1:1). After vigorous stirring for 1 h, the mixture was then transferred into an autoclave and heated at 200 °C for 48 h. The ANP_mr_ products obtained at 200 °C for 24 and 36 h duration were also tested for comparison. The resulted greenish yellow precipitates were thoroughly washed with deionised water and ethanol to remove any unreacted materials. Finally, the powder was dried slowly at 50 °C in oven or left in air to dry at room temperature overnight. The microrod-like product (evident from SEM and TEM images) is abbreviated herein as ANP_mr_. The control experiments were also performed using ethylene glycol alone or water alone at the same time period of 48 h and constant temperature of 200 °C. From SEM and TEM, the product from water alone gave platelet-like morphology (abbreviated herein as ANPmp), while that from ethylene glycol alone gave dendrite-like morphology (abbreviated herein as ANP_md_).

The formation of NH_4_NiPO_4_.H_2_O was investigated by PANalytical X’Pert PRO diffractometer equipped with Ni-filtered Cu K-alpha radiation (λ = 1.541841 A). The morphology of the as-synthesized powders was analysed using JEOL- JSM 7500F scanning electron microscope operated at 2.0 kV. TEM and HRTEM images were obtained from JEOL-Jem 2100 microscope operated at an acceleration voltage of 200 kV. BET measurements were performed to measure the specific surface area and pore size based on the N_2_ adsorption–desorption method by using Micromeritics TriStar II instrument.

### Materials, preparation and pseudocapacitor fabrication

Nickel foam (Celmet: thickness = 1.6 mm, surface area 7500 m^2^, cell size = 0.5 mm, 48–52 cells per inch) and carbon cloth (B-1/C, E-TEK) were used as substrates and current collectors in the fabrication of the half-cell (three-electrode system) and full-cell (two-electrode systems), respectively. Prior to use, the nickel foam was properly cleaned as we described before^39^ by first sonicating in 1 M HCl solution for 30 min, washed several times with copious amount of de-ionized water, and then dried under vacuum. The electrolyte materials, polyvinyl alcohol (PVA), potassium hydroxide (KOH) and sodium sulphate (Na_2_SO_4_) were procured from Sigma-Aldrich and used as received. For the three-electrode system, the electrode materials were prepared by coating a slurry mixture of NH_4_NiPO_4_.H_2_O (ANP), carbon black and polyvinylidene fluoride (PVDF) (80:15:5 weight ratio) on a piece of clean nickel foam and dried overnight in a vacuum oven at 80 °C. The mass of the active material on nickel foam was 0.32 mg for ANP_mr_, 0.35 mg for ANP_md_ and 0.34 mg for ANP_mp_. For the symmetric and asymmetric systems, the electrode materials were prepared by coating a slurry mixture of ANP_mr_, activated carbon (AC, Norit^®^ supra 30) and PVDF (50:40:10 weight ratio) on carbon cloth (disc = 1.6 cm^2^). The symmteric (ANP_mr_//ANP_mr_) and asymmetric (ANP_mr_//AC) pseudocapacitors were performed in 3 M KOH and 1 M Na_2_SO_4_, respectively. The all-solid-state flexible symmetric pseudocapacitor (square = 1 cm^2^) was fabricated in a similar manner as the symmetric cells but using a polymer gel electrolyte (PVA-KOH). In a typical polymer electrolyte preparation, 8 g of PVA and 4 g of KOH were dissolved in deionised water (40 ml) and the mixture was stirred at 90 °C for 1 h to form a gel. Various forms of the ANP systems (ANP_mp_, ANP_md_, ANP_mr_ as well the ANP_mr_ obtained at different 24 and 36 h at the same temperature) were investigated as possible candidates as all-solid-state flexible pseudocapacitors.

### Electrochemical measurements and calculations

All electrochemical tests involving cyclic voltammetry (CV), electrochemical impedance spectroscopy (EIS) and galvanostatic charge-discharge analysis (including voltage-holding experiments) were performed at room temperature using computer-controlled Multi-channel Potentiostat/Galvanostat Bio-Logic VMP3 work station driven by EC-Lab^®^ v10.40 software with Z-fit tool for EIS data analysis. In a typical three-electrode system, ANP-coated nickel foam was used as the working electrode, platinum mesh as the counter electrode, and Ag/AgCl (3 M KCl) as the reference electrode, in 3 M KOH aqueous solution. EIS measurements were carried out in the frequency ranging from 10 kHz to 10 mHz at the open circuit voltage with AC voltage amplitude of 1.5 mV.

For the half-cells (3-electrode configurations), the specific capacitance (*C*_sp_), maximum specific power density (*P*_max_) and specific energy density (*E*_sp_) was evaluated using the conventional equations (2–4)^40^:


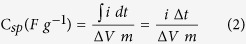



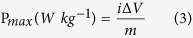



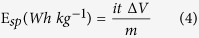


where: *i* (A) is the current, Δ*V* (V)/Δ*t* (s) the slope of the discharge curve, and *m* (g) the mass of active materials (ANP and carbon), *V* (V) is the voltage obtained during charge. For the full cells (2-electrode configurations), the corresponding parameters were obtained using conventional equations (5, 6, 7, 8, 9):


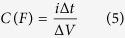



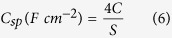



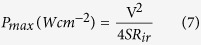


where,


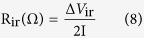



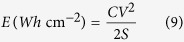


where *i* (A) is the applied current, Δ*V* (V)/Δ*t* (s) the slope of the discharge curve and *S* (cm^−2^) the total geometric surface area of the two electrodes, *C* (F) the calculated capacitance, *V* (V) is the maximum voltage obtained during charge, and *R*_*ir*_ is the internal resistance which is determined from the voltage drop at the beginning of each discharge, while the Δ*V*_*ir*_ represents the voltage drop.

## Additional Information

**How to cite this article**: Raju, K. and Ozoemena, K. I. Hierarchical One-Dimensional Ammonium Nickel Phosphate Microrods for High-Performance Pseudocapacitors. *Sci. Rep.* **5**, 17629; doi: 10.1038/srep17629 (2015).

## Supplementary Material

Supplementary Information

## References

[b1] Simon P. & Gogosti Y. Materials for electrochemical capacitors. Nat. Mater. 7, 845–854 (2008). 18956000 10.1038/nmat2297

[b2] Miller J. R. & Simon P. Electrochemical capacitors for energy management. Science 321, 651–652 (2008). 18669852 10.1126/science.1158736

[b3] Arico A. S., Bruce P., Scrosati B., Tarascon J.-M. & Van Schalkwijk W. Nanostructured materials for advanced energy conversion and storage devices. Nat. Mater. 4, 366–377 (2005). 15867920 10.1038/nmat1368

[b4] El Kady M. F., Strong V., Dubin S. & Kaner R. B. Laser scribing of high-performance and flexible graphene-based electrochemical capacitors. Science 335, 1326–1330 (2012). 22422977 10.1126/science.1216744

[b5] Naoi K., Naoi W., Aoyagi S., Miyamoto J.-I. & Kamino T. New generation “nanohybrid supercapacitor”. Acc. Chem. Res. 46, 1075–1083 (2013). 22433167 10.1021/ar200308h

[b6] Simon P., Taberna P.-L. & Beguin F. in Supercapacitors: Materials, Systems and Applications (ed. Beguin F. & Frackowiak E. ) 131–165 (Wiley-VCH, 2013).

[b7] Wang G. P., Zhang L. & Zhang J. A review of electrode materials for electrochemical supercapacitors. Chem. Soc. Rev. 41, 797–828 (2012). 21779609 10.1039/c1cs15060j

[b8] Zhang L. L. & Zhao X. S. Carbon-based materials as supercapacitor electrodes. Chem. Soc. Rev. 38, 2520–2531 (2009). 19690733 10.1039/b813846j

[b9] Lu X., Yu M., Wang G., Tong Y. & Li Y. Flexible solid-state supercapacitors: design, fabrication and applications. Energy Environ. Sci. 7, 2160–2181 (2014).

[b10] Yang P. & Mai W. Flexible solid-state electrochemical supercapacitors. Nano Energy 8, 274–290 (2014).

[b11] Zhao X., Sanchez B. M., Dobson P. J. & Grant P. S. The role of nanomaterials in redox-based supercapacitors for next generation energy storage devices. Nanoscale, 3, 839–855 (2011). 21253650 10.1039/c0nr00594k

[b12] Peng X., Peng L., Wu C. & Xie Y. Two dimensional nanomaterials for flexible supercapacitors. Chem. Soc. Rev. 43, 3303–3323 (2014). 24614864 10.1039/c3cs60407a

[b13] Zhao J. *et al.* Mesoporous uniform ammonium nickel phosphate hydrate nanostructures as high performance electrode materials for supercapacitors. Cryst Eng Comm. 15, 5950–5955 (2013).

[b14] Wang S. *et al.* NH_4_CoPO_4_·H_2_O microbundles consisting of one-dimensional layered microrods for high performance supercapacitors. RSC Adv. 4, 340–347 (2014).

[b15] Wu C. *et al.* Two-dimensional vanadyl phosphate ultrathin nanosheets for high energy density and flexible pseudocapacitors. Nature. Commun. 4, 2431 (2013). 24026224 10.1038/ncomms3431

[b16] Ahn Y. R., Park C. R., Jo S. M. & Kim D. Y. Enhanced charge-discharge characteristics of RuO_2_ supercapacitors on heat-treated TiO_2_ nanorods. Appl. Phys. Lett. 90, 122106 (2007).

[b17] Teng F. *et al.* Self-assembly of LiFePO_4_ nanodendrites in a novel system of ethylene glycol–water. J. Crystal Growth. 312, 3493–3502 (2010).

[b18] Ma G. M., Zhu Y.-J. & Chang J. Monetite formed in mixed solvents of water and ethylene glycol and its transformation to hydroxyapatite. J. Phys. Chem. B, 110, 14226–14230 (2006). 16854124 10.1021/jp061738r

[b19] Carling S. G., Day P. & Vissen D. Crystal and magnetic structures of layer transition metal phosphate hydrates. Inorg. Chem. 34, 3917–3927 (1995).

[b20] Fang Y. P. *et al.* Systematic synthesis and characterization of single-crystal lanthanide orthophosphate nanowires. J. Am. Chem. Soc. 125, 16025–16034 (2003). 14677994 10.1021/ja037280d

[b21] Yang C., Lei Dong L., Chen Z. & Lu H. High-performance all-solid-state supercapacitor based on the assembly of graphene and manganese (II) phosphate nanosheets. J. Phys. Chem. C, 118, 18884–18891 (2014).

[b22] Zang J. & Li X. *In situ* synthesis of ultrafine β-MnO_2_/polypyrrole nanorod composites for high-performance supercapacitors. J. Mater. Chem. 21, 10965 –10969 (2011).

[b23] Perera S. D. *et al.* Vanadium oxide nanowire – Graphene binder free nanocomposite paper electrodes for supercapacitors: A facile green approach. J. Power Sources 230, 130–137 (2013).

[b24] Zhou W. *et al.* One-step synthesis of Ni_3_S_2_ nanorod@Ni(OH)_2_ nanosheet core–shell nanostructures on a three-dimensional graphene network for high-performance supercapacitors. Energy Environ. Sci. 6, 2216–2221 (2013).

[b25] Pech D. *et al.* Ultrahigh-power micrometre-sized supercapacitors based on onion-like carbon. Nat. Nanotech. 5, 651–654 (2010). 10.1038/nnano.2010.162 20711179

[b26] Lee J. A. *et al.* Ultrafast charge and discharge biscrolled yarn supercapacitors for textiles and microdevices. Nature Commun. 4, 1970 (2013). 23733169 10.1038/ncomms2970

[b27] Wang C., Zhan Y., Wu L., Li Y. & Liu J. High-voltage and high-rate symmetric supercapacitor based on MnO_2_ -polypyrrole hybrid nanofilm. Nanotechnology 25, 305401 (2014). 25008287 10.1088/0957-4484/25/30/305401

[b28] Padmanathan N., Selladurai S. & Razeeb K. M. Ultra-fast rate capability of a symmetric supercapacitor with a hierarchical Co_3_O_4_ nanowire/nanoflower hybrid structure in non-aqueous electrolyte. RSC Adv. 5, 12700–12709 (2015).

[b29] Yang P. H. *et al.* Hydrogenated ZnO core-shell nanocables for flexible supercapacitors and self-powered systems. ACS Nano 7, 2617 –2626 (2013). 23368853 10.1021/nn306044d

[b30] Lu X. *et al.* H-TiO_2_ @MnO _2_//H-TiO _2_ @C Core–Shell nanowires for high performance and flexible asymmetric supercapacitors. Adv. Mater. 25, 267–272 (2013). 23080535 10.1002/adma.201203410

[b31] Kim J.-H., Zhu K., Yan Y., Perkins C. L. & Frank A. J. Microstructure and pseudocapacitive properties of electrodes constructed of oriented NiO-TiO_2_ nanotube arrays. Nano Lett. 10, 4099–4104 (2010). 20873847 10.1021/nl102203s

[b32] Li R., Ren X., Zhang F., Du C. & Liu J. Synthesis of Fe_3_O_4_@SnO_2_ core–shell nanorod film and its application as a thin-film supercapacitor electrode. Chem. Commun. 48, 5010–5012 (2012). 10.1039/c2cc31786a 22510855

[b33] Lu X. H. *et al.* WO_3−x_@Au@MnO_2_ core-shell nanowires on carbon fabric for high-performance flexible supercapacitors. Adv Mater. 24, 938–944 (2012). 22403832 10.1002/adma.201104113

[b34] Yu G. H. *et al.* Solution-processed graphene/MnO_2_ nanostructured textiles for high-performance electrochemical capacitors. Nano Lett. 11, 2905–2911 (2011). 21667923 10.1021/nl2013828

[b35] Pech D. *et al.* Elaboration of a microstructured inkjet-printed carbon electrochemical capacitor. J. Power Sources 195, 1266–1269 (2010).

[b36] Xu Y. *et al.* Flexible solid-state supercapacitors based on three-dimensional graphene hydrogel films. ACS Nano. 7, 4042–4049 (2013). 23550832 10.1021/nn4000836

[b37] Yang P. *et al.* Hydrogenated ZnO coreshell nanocables for flexible supercapacitors and self-powered systems. ACS Nano. 7, 2617–2626 (2013). 23368853 10.1021/nn306044d

[b38] Fu Y. P. *et al.* Fiber supercapacitors utilizing pen ink for flexible/wearable energy storage. Adv. Mater. 24, 5713–5718 (2012). 22936617 10.1002/adma.201202930

[b39] Makgopa K. *et al.*, A high-rate aqueous symmetric pseudocapacitor based on highly graphitized onion-like carbon/birnessite-type manganese oxide nanohybrids, J. Mater. Chem. A. 3, 3480–3490 (2015).

[b40] Stoller M. D. & Ruoff R. S. Best practice methods for determining an electrode material’s performance for ultracapacitors. Energy Environ. Sci. 3, 1294–1301 (2010).

