# Supplementary material for: Hierarchical One-Dimensional Ammonium Nickel Phosphate Microrods for High-Performance Pseudocapacitors
Source: Sci Rep. 2015 Dec 3;5:17629. doi: 10.1038/srep17629 (PMC4668563; doi:10.1038/srep17629)
Supplement: Supplementary Information [file srep17629-s1.doc]

**SUPPLEMENTARY MATERIALS**

**Hierarchical One-Dimensional Ammonium Nickel Phosphate Microrods for High-Performance Pseudocapacitors**

**Kumar Raju^1^ and Kenneth I. Ozoemena1,2[[1]](#footnote-2)***

^1^Energy Materials Unit, Materials Science and Manufacturing, Council for Scientific & Industrial Research (CSIR), Pretoria 0001, South Africa

^2^School of Chemistry, University of the Witwatersrand, Johannesburg 2050, South Africa

**Figure-S1 (Ozoemena):** Comparative morphologies from SEM (left images) and TEM (right images) of ANP_mr_ prepared at 24, 36 and 48 h.


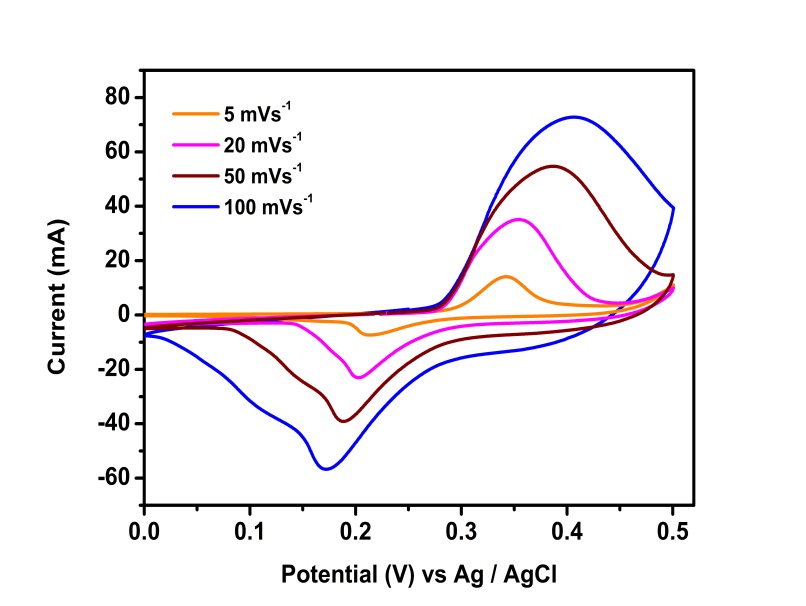


a


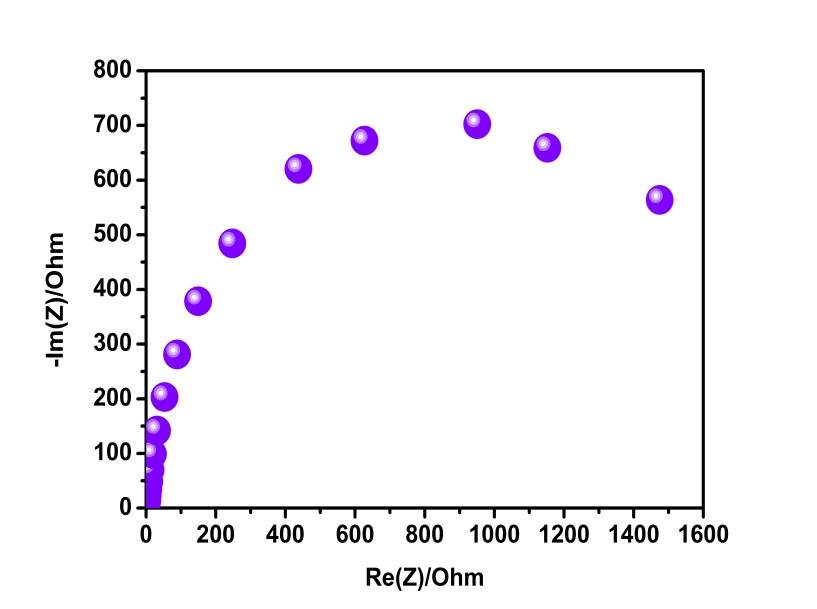


b


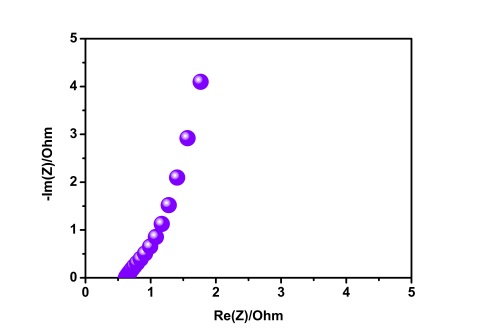


**Figure-S2 (Ozoemena):** (a) CV curves of ANP_mr_ at different scan rates (b) Nyquist plot of ANP_mr_ after 5,000 consecutive cycles. Experimental conditions: Nickel foam as the working electrode; 3M KOH as the aqueous electrolyte. Figure (c) represents the electrical equivalent circuit (Voigt circuit) used in fitting the pseudocapacitors investigated in this work.

**Table-S1 (Ozoemena):** Specific capacitance, energy and power density of ANP_mr_ electrode compared with other related pseudocapactive materials in *three-electrode* (half-cell system.

| Material | Electrolyte | Specific capacitance (F g^-1^) | Maximum Energy density  (W h kg^-1^) | Maximum Power density  (kW kg^-1^) | Ref |
| --- | --- | --- | --- | --- | --- |
| NH_4_NiPO_4_.H_2_O microrods | 3 M KOH | 1311 @ 1 A g^-1^ | 65.78 | 21.25 | This work |
| NH_4_NiPO_4_.H_2_O nanoalmond | 3 M KOH | 1072 @ 1.5 A g^-1^ | 30.2 | 2.82 | 1 |
| NH_4_CoPO_4_.H_2_O nano/microstructures | 3 M KOH | 369.4 @ 0.625A g^-1^ | 10.4 | 1.407 | 2 |
| NH_4_CoPO_4_.H_2_O microbundles/graphene | 3 M KOH | 662 @ 1.5 A g^-1^ | 26.6 | 0.852 | 3 |
| Ni-Co hydroxide nanorods | 1M KOH | 456 @ 20 mV s^-1^ | 12.8 | - | 4 |
| Mesoporous Ni_0.3_Co_2.7_O_4_ | 3M KOH | 960 @ 0.625 A g^-1^ | 141 | 27.1 | 5 |
| Phosphate -carbon nanotube | 6 M KOH | ~158 @ 0.1 A g^-1^ | 8.2 | 0.03 | 6 |
| Ni_3_S_2_ nanorod/  Ni(OH)_2_/graphene | 3 M KOH | 1037.5 @ 5.1 A g^-1^ | 70.6 | 5 | 7 |

**Table-S2 (Ozoemena): Comparative fitting parameters for the electrochemical impedance spectroscopy (EIS) data of the ANP-based pseudocapacitor systems using the Voigt electrical equivalent circuit (Figure-2S (c)).**

| **Systems** | **Electrochemical impedance spectroscopy parameters** | | | | | | |
| --- | --- | --- | --- | --- | --- | --- | --- |
|  | ***R_s_/Ω*** | ***Q_1_ / µF.s^(α-1)^*** | ***R_ct1_ /Ω*** | ***n_1_*** | ***Q_2_ / mF.s^(α-1)^*** | ***R_ct2_ /Ω*** | ***n_2_*** |
| ***Three electrode systems*** | | | | | | | |
| ANPmp | 1.48±0.14 | 3.45±0.12 | 26.35±0.59 | 0.82±0.13 | 37.31±2.85 | 15.02±0.1232 | 0.85±0.21 |
| ANPmd | 1.72±0.24 | 2.88±1.25 | 5.19±0.53 | 0.81±0.24 | 1.062±0.21 | 26.19±0.1415 | 0.82±0.20 |
| ANPmr | 0.79±0.21 | 0.82±0.14 | 23.82±13.10 | 0.58±0.27 | 7.438±3.88 | 1.275±0.113 | 0.53±0.27 |
| ***Symmetric /Asymmetric systems*** | | | | | | | |
| ANPmr symmetric | 0.13±0.08 | 0.33±0.08 | 32.26±5.44 | 0.82±0.35 | 1.05±1.26 | 0.20±0.11 | 0.87±0.15 |
| ANPmr asymmetric | 0.55±0.04 | 2.22±0.62 | 38.75±5.96 | 0.87±0.15 | 26.61±7.55 | 2.47±0.25 | 0.82±0.24 |
| ***All-solid-state pseudocapacitors*** | | | | | | | |
| ANPmp | 10.8±1.37 | 15.56±6.14 | 77.01±6.02 | 0.67±0.33 | 3.05±1.04 | 16.40±9.55 | 0.83±0.42 |
| ANPmd | 24.5±4.04 | 22.79±1.43 | 67.74±11.30 | 0.81±0.27 | 4.43±0.78 | 47.00±3.86 | 0.81±0.32 |
| ANPmr | 4.59±0.83 | 4.61±0.12 | 13.78±1.20 | 0.86±0.31 | 2.81±0.97 | 4.95±0.742 | 0.80±0.26 |
| ANPmr @ 36 h | 4.51±1.94 | 4.90±0.38 | 17.86±1.13 | 0.88±0.21 | 8.61±0.60 | 122.50±11.29 | 0.51±0.14 |
| ANPmr @ 24 h | 4.62±0.23 | 4.05±0.36 | 19.75±8.80 | 0.72±0.33 | 19.75±2.6 | 11.71±1.96 | 0.83±0.51 |


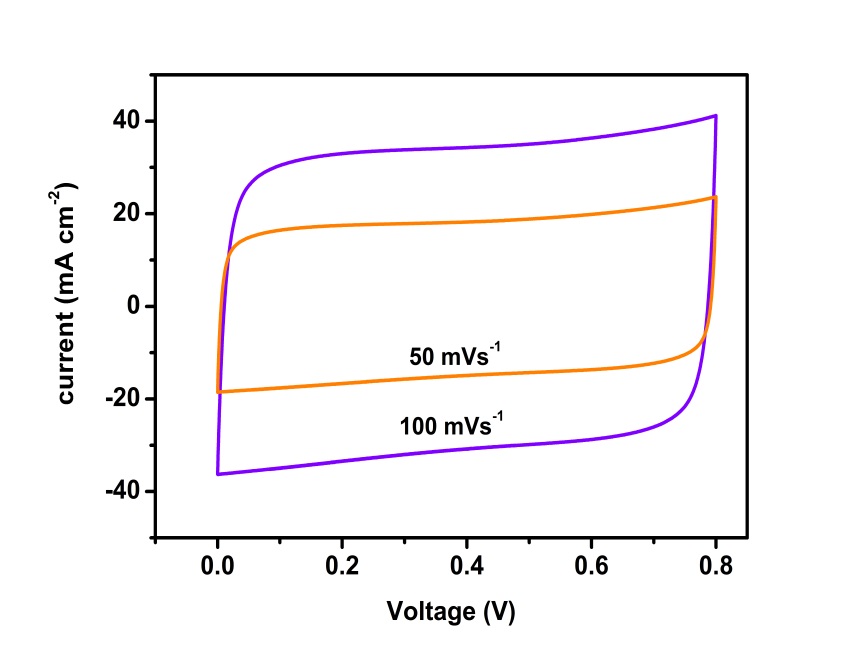


a


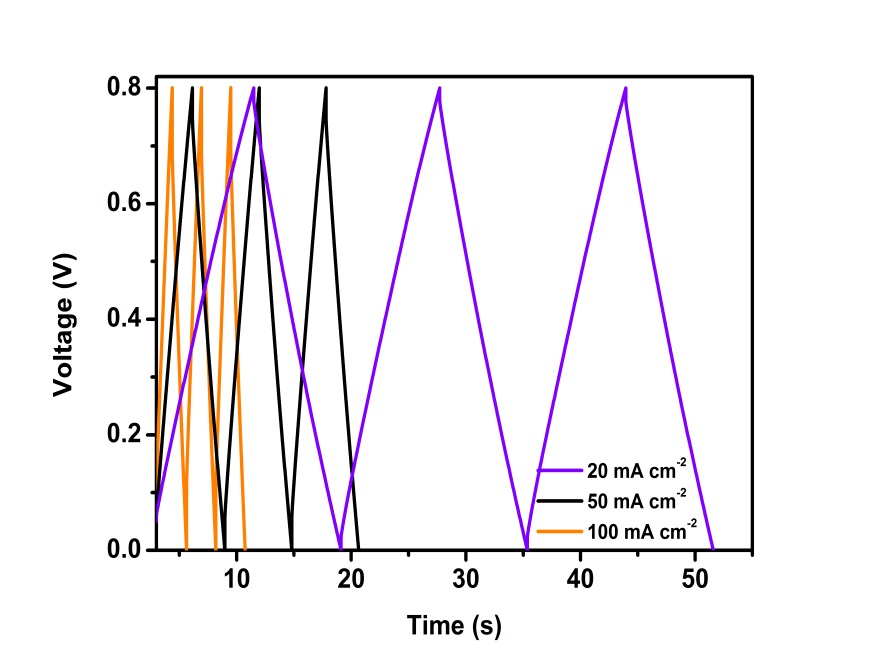


b

Fig.S5

**Figure-S3 (Ozoemena):** CV curves of ANP_mr_ symmetric pseudocapacitor at scan rates of 50 and 100 mVs^-1^ and (b) Charge –discharge curves of ANP_mr_ symmetric at different current densities. Experimental conditions: Carbon cloth as substrate, and 3M KOH as electrolyte.

**Figure-S4 (Ozoemena):** Typical electrochemical data for all solid-state flexible ANP_mr_-based symmetric pseudocapacitor fabricated on nickel foam as substrate/current collector in PVA/KOH polymer electrolyte: (a) CV curves at scan rates of 5 – 100 mVs^-1^, (b) Charge –discharge curves at different current densities, (c) areal capacitance at different current densities, and (d) Nyquist plot at OCV. Note that the redox peaks in the 3-electrode configuration (Figure-S2 above) disappeared in this 2-electrode configuration. We do not fully understand the reason, but it seems that in 3-electrode system, the redox species have greater access to the electrolyte and can displays their redox peaks easily than when deployed in 2-electrode systems. **N**ickel foam plays no significant role in the electrochemistry of the 3-electrode. Infact, we have made similar observation in our previous studies^8^ (please see ESI Fig. 3 of Makgopa et al., *J. Mater. Chem. A,* 2015, 3, 3480–3490) that Nickel foam plays no significant role other than a good current conductor.


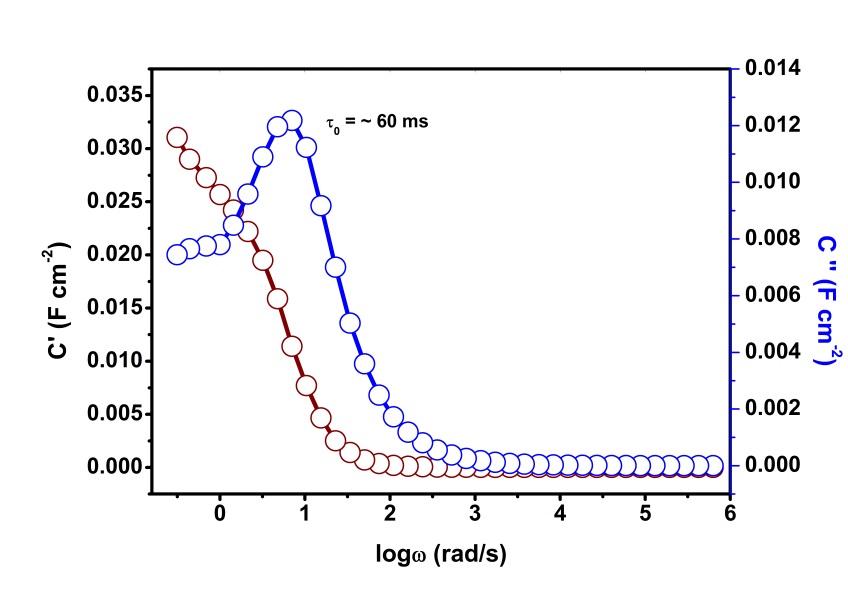

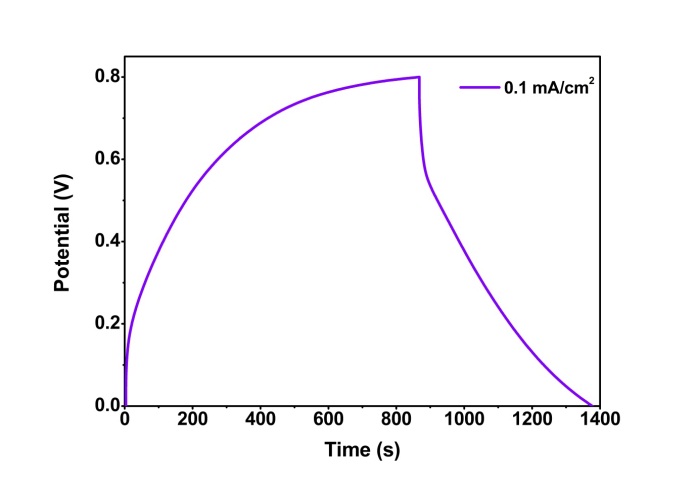


f

b


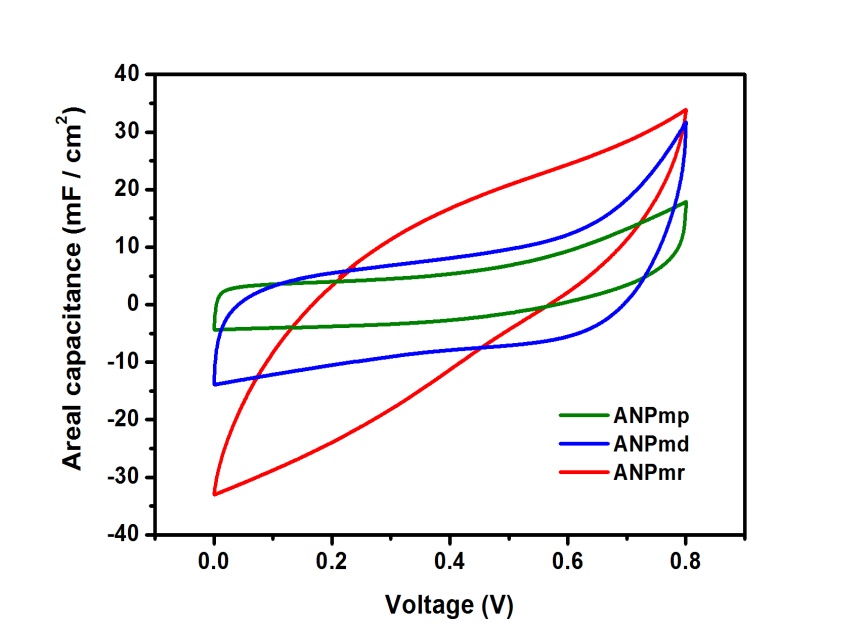


a


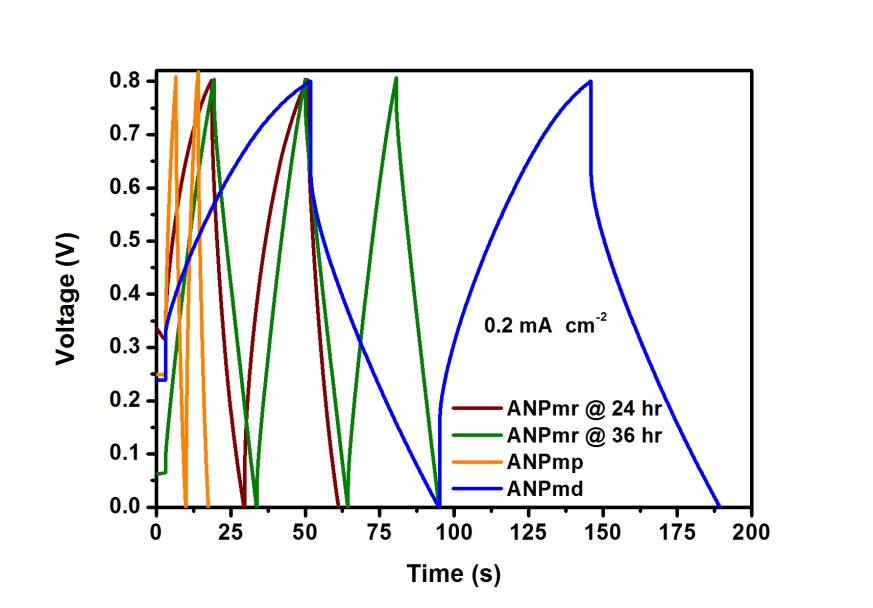


c


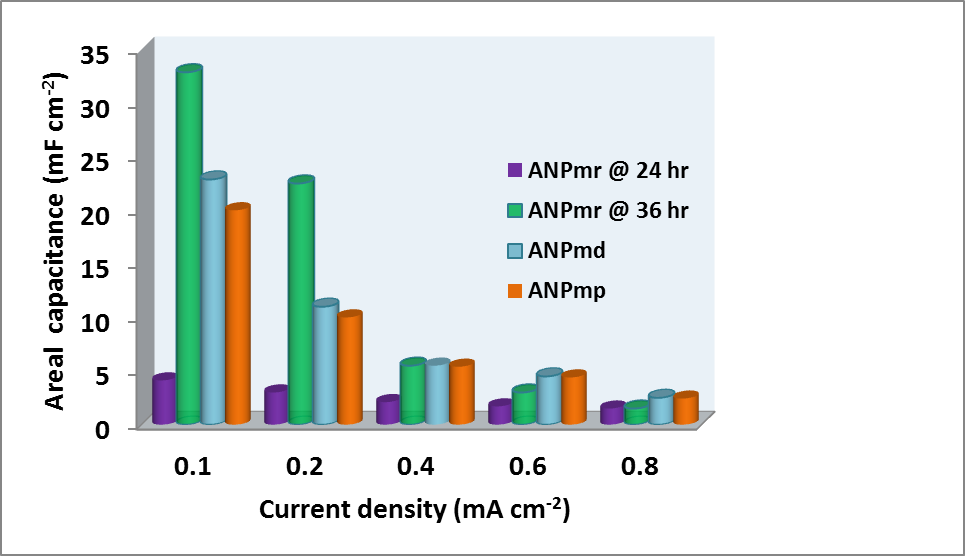


d


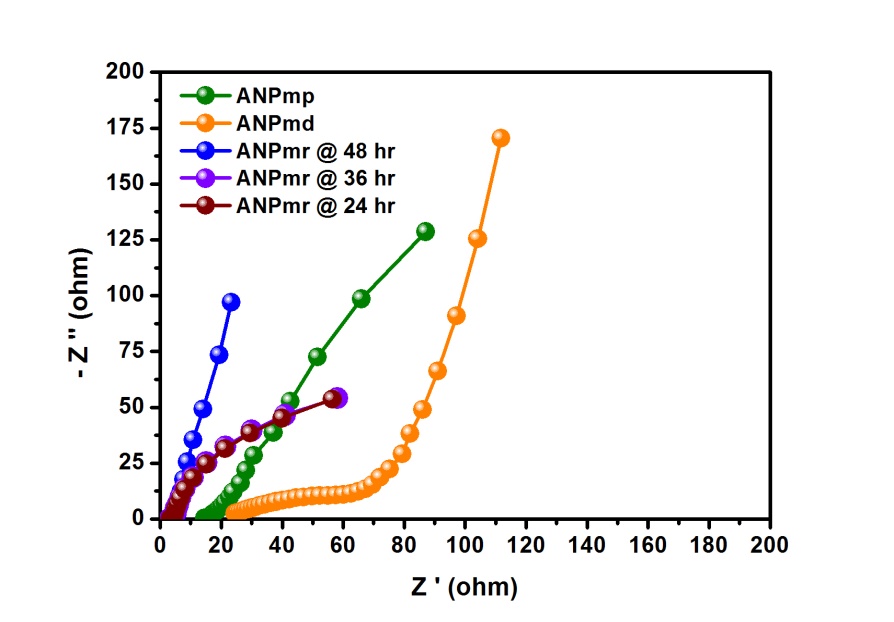


e


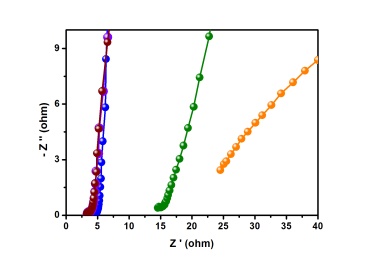


**Figure-S5 (Ozoemena):** Comparative electrochemical performances of different all-solid-state flexible symmetric pseudocapacitors fabricated on a carbon cloth with PVA/KOH polymer electrolyte: (a) Areal capacitance calculated from CV curves at a scan rate of 10 mVs^-1^; (b) Charge –discharge profile of ANP_mr_ (48 h) at 0.1 mA cm^-2^; (c) Charge –discharge profiles of ANP electrodes at 0.2 mA cm^-2^; (d) Areal capacitance of ANP_md_, ANP_mp_ and ANP_mr_ electrodes prepared at 24 and 36 h against different current densities; (e) Nyquist plots of ANP electrodes, insert shows the magnified view at high frequencies; and (f) Frequency dependence of the real and imaginary parts of areal capacitance of ANP_mr_ (48 h) electrode. Note that the areal capacitance values of ANP_mr@24h_ and ANP_mr@36h_ are lower than those of the ANP_md_ and ANP_mp_ at 0.4 – 0.8 mA cm^-2^. This behaviour is very much opposite to the microrods that was obtained at the optimized reaction time of 48 h (ANP_mr_). Thus, we can attribute this behaviour to the poorly grown microrods at the 24 – 36 h, which limits both the surface area and pores on the surface thereby leading to the poor rate capability (i.e., lower capacitance at higher current density).


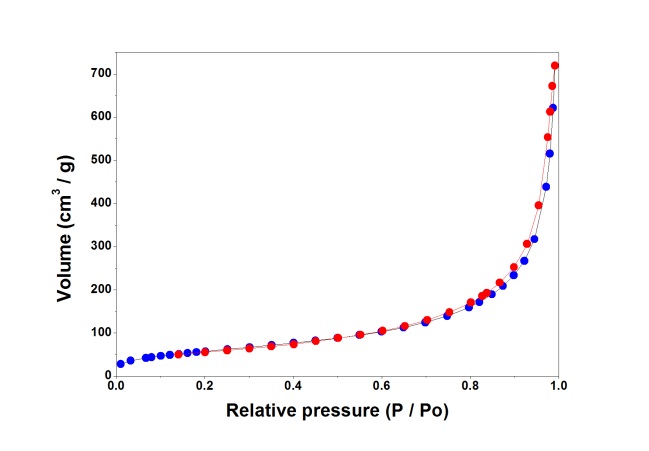


a


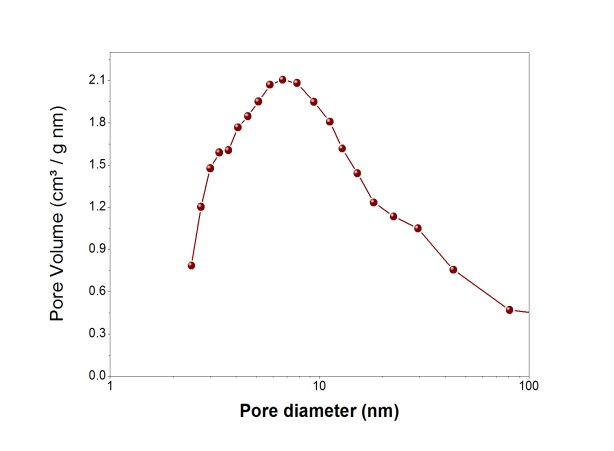


b

**Figure-S6 (Ozoemena):** (a) Nitrogen adsorption-desorption isotherms and (b) pore size distribution profile of ANP_mr_ prepared at 48 h.


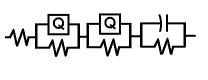

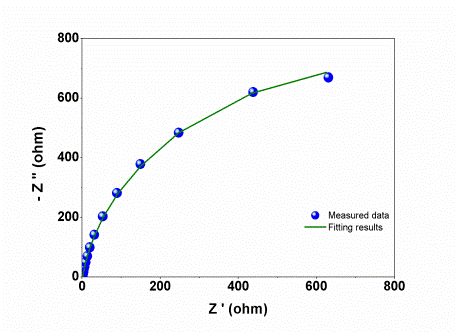

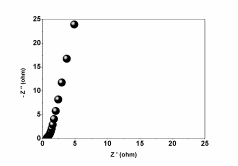


| **Material** | **Surface area (m^2^/g)** |
| --- | --- |
| ANP_mr_ (48 h) | 214 |

**Table-S3 (Ozoemena):** Areal capacitance values of symmetric /asymmetric supercapacitor of ANP_mr_ compared with literature values of other symmetric /asymmetric supercapacitors.

| **Materials** | **Electrolyte** | **Specific capacitance** | **Reference** |
| --- | --- | --- | --- |
| ANP_mr_ //ANP_mr_  ANP_mr_ //AC  ZnO nanowire-MnO_2_ | 3 M KOH  1 Na_2_SO_4_  1 M KNO_3_ | 138 mF cm^-2^ @ 20 mA cm^-2^  221 mF cm^-2^ @ 20 mA cm^-2^  0.21mFcm^-2^ @100mVs^-1^ | This work  This work  9 |
| MnO_2_-polypyrrole hybrid | 1 M Li_2_SO_4_ | 25.9 mFcm^-2^ | 10 |
| MnO_2_//MnO_2_ | 0.5M Na_2_SO_4_ | 26 mFcm^-2^ @0.5 mAcm^-2^ | 11 |
| Co_3_O_4_ nanowire/flower | 3 M KOH | 7.8 mFcm^−2^ | 12 |
| Ru//Ru | 1 M Na_2_SO_4_ | 67 mFcm^-2 @^ 1 mAcm^−2^ | 13 |
| Carbon // carbon | EMIM][NTf_2_] | 32 mFcm^−2^ | 14 |
| H-TiO_2_ @ MnO_2_ | 5 M LiCl | 0.9 Fcm^-3^ | 15 |
| WO_3_-x@Au@MnO_2_  core–shell nanowires | 0.1 M Na_2_SO_4_ | 57 mFcm^-2^ | 16 |
| Graphene/CNT/Fe_3_O_4_ | 1M Na_2_SO_4_ | 0.98 mFcm^-2^ | 17 |
| Graphene + MnO_2_ | 0.5 M Na_2_SO_4_ | 275 mFcm^-2^ @ 5mVs^-1^ | 18 |
|  |  |  |  |

**Table-S4 (Ozoemena):** Performance of all solid-state flexible symmetric supercapacitor of ANP_mr_ electrode compared with other supercapacitors fabricated in all solid-state method.

| Materials | Electrolyte | Areal capacitance (mF cm^-2^) | Energy density (mWh cm^-2^) | Power density (mWcm^-2^) | Ref |
| --- | --- | --- | --- | --- | --- |
| ANP_mr_ | PVA/KOH | 66 | 21.2 | 12.7 | This work |
| CNTS | PVA/H_3_PO_4_ | 7.34 | - | - | 19 |
| Graphene | PVA/H_3_PO_4_ | 3.67 | - | - | 20 |
| Pen Ink | PVA/H_2_SO_4_ | 19.5 | 2.70x10^-3^ | 9.07 | 21 |
| ZnO nanowire- MnO_2_ coated | PVA/H_3_PO_4_ | 2.24 | 2.78 x 10^-5^ | 14 | 9 |
| GF is covered with 3D porous graphene (GF@3D-G) | PVA/H_2_SO_4_ | 1.7 | 1.7x10^-1^ | 100 | 22 |
| β-Ni(OH)_2_/Graphene Nanohybrids | PVA/H_3_PO_4_ | 3.34 | - | - | 23 |
| Graphene and Manganese (II) Phosphate Nanosheets | PVA / KOH | 40 | 0.17x10^-9^ | 46x10^-9^ | 24 |
| Two dimensional vanadyl phosphate ultrathin nanosheets | PVA/LiCl | 8.3 | 1.7 | 5.2 | 25 |
| ZnO core-shell nanocables | PVA/LiCl | 26 | 0.04 | 2.44 | 26 |
| VS_2_ nanosheets | PVA /BMIMBF_4_ | 4.76 | - | - | 27 |

**References**

1. Zhao, J. et al. Mesoporous uniform ammonium nickel phosphate hydrate nanostructures as high performance electrode materials for supercapacitors. *CrystEngComm.* **15,** 5950-5955 (2013)
2. Pang, H., Yan, Z., Wang, W., Chen, J., Zhang, J. & Zheng, H. Facile fabrication of NH_4_CoPO_4_.H_2_O nano/microstructures and their primarily application as electrochemical supercapacitor. *Nanoscale* **4**, 5946 -5953 (2012).
3. Zang, J. & [Li](http://pubs.rsc.org/en/results?searchtext=Author%3AXiaodong Li), X.  In situ synthesis of ultrafine β-MnO_2_/polypyrrole nanorod composites for high-performance supercapacitors. *J. Mater. Chem.* **21,** 10965 -10969 (2011).
4. Salunkhe, R. R.,  Jang, K., Lee, S-W. & Ahn, H. Aligned nickel-cobalt hydroxide nanorod arrays for electrochemical pseudocapacitor applications. *RSC Adv*. **2**, 3190 -3193 (2012).
5. Perera, S.D. et al. Vanadium oxide nanowire – Graphene binder free nanocomposite paper electrodes for supercapacitors: A facile green approach. *J. Power Sources* **230**, 130-137 (2013).
6. Fan, X.**,Yu, C., Ling, Z., Yang, J. & Qiu**, J. Hydrothermal synthesis of phosphate-functionalized carbon nanotube-containing carbon composites for supercapacitors with highly stable performance. *ACS Appl. Mater. Interfaces* **5**, 2104 -2110 (2013).
7. Zhou, W. et al. One-step synthesis of Ni_3_S_2_ nanorod@Ni(OH)_2_nanosheet core–shell nanostructures on a three-dimensional graphene network for high-performance supercapacitors. *Energy Environ. Sci.* **6**, 2216-2221 (2013).
8. Makgopa, K. et al., A high-rate aqueous symmetric pseudocapacitor based on highly graphitized onion-like carbon/birnessite-type manganese oxide nanohybrids, *J. Mater. Chem. A.* **3**, 3480–3490 (2015).
9. Bae, J. et al., Fiber supercapacitors made of nanowire-fiber hybrid structures for wearable/flexible energy storage. *Angew. Chem. Int. Ed.* **50**, 1683 –1687 (2011).
10. Wang, C., Zhan, Y., Wu, L., Li, Y & Liu, J. High-voltage and high-rate symmetric supercapacitor based on MnO_2_ -polypyrrole hybrid nanofilm. *Nanotechnology* **25,** 305401 (2014).
11. Yang, P. H. et al. Hydrogenated ZnO core-shell nanocables for flexible supercapacitors and self-powered systems. *ACS Nano* **7**, 2617 -2626 (2013).
12. Padmanathan, N., Selladurai, S. &  Razeeb, K.M.  Ultra-fast rate capability of a symmetric supercapacitor with a hierarchical Co3O4 nanowire/nanoflower hybrid structure in non-aqueous electrolyte. *RSC Adv.* **5,** 12700-12709 (2015).
13. Xia, H. Bo Li, B. &   Lu, L. 1.8 V symmetric supercapacitors developed using nanocrystalline Ru films as electrodes. *RSC Adv.* **4,** 11111- 11114 (2014).
14. Kang, Y.J., Chung, H., Han, C.H. & Kim, W. All-solid-state flexible supercapacitors based on papers coated with carbon nanotubes and ionic-liquid-based gel electrolytes. *Nanotechnology* **23,** 065401 (2012).
15. Lu, X. et al. H-TiO _2_ @MnO _2_ //H-TiO _2_ @C Core–Shell nanowires for high performance and flexible asymmetric supercapacitors. *Adv. Mater.* **25,** 267-272 (2013).
16. Lu, X. H. et al. WO_3_−x@Au@MnO_2_ core-shell nanowires on carbon fabric for high-performance flexible supercapacitors. *Adv Mater.* **24,** 938-944 (2012).
17. Cheng, H. H. et al. Textile electrodes woven by carbon nanotube–graphene hybrid fibers for flexible electrochemical capacitors. *Nanoscale* **5,** 3428- 3434 (2013).
18. Yu, G. H. et al. Solution-processed graphene/MnO_2_ nanostructured textiles for high-performance electrochemical capacitors. *Nano Lett.* **11**, 2905-2911 (2011).
19. Kaempgen, M., **Candace K. Chan, C.K., Ma, J., Cui, Y. & Gruner, G.** Printable thin film supercapacitors using single-walled carbon nanotubes. *Nano Lett.* **9,** 917–922 (2009).
20. El Kady, M. F. et al. Laser scribing of high-performance and flexible graphene-based electrochemical capacitors. *Science* **335**, 1326-1330 (2012).
21. Fu, Y. P. et al. Fiber supercapacitors utilizing pen ink for flexible/wearable energy storage, *Adv. Mater.* **24,** 5713–5718 (2012).
22. Meng, Y. et al., All-graphene core-sheath microfibers for all-solid-state, stretchable fibriform supercapacitors and wearable electronic textiles. *Adv. Mater*. **25**, 2326–2331 (2013).
23. Xie, J. et al. Layer-by-layer β-Ni(OH)_2_/graphene nanohybrids for ultraflexible all-solid-state thin-film supercapacitors with high electrochemical performance. *Nano Energy* **2,** 65–74 (2013).
24. Yang, C., Lei Dong, L.,  Chen, Z &  Lu, H. High-performance all-solid-state supercapacitor based on the assembly of graphene and manganese (II) phosphate nanosheets. *J. Phys.Chem. C,* ***118*,** 18884–18891 (2014).
25. Wu, C. et al. Two-dimensional vanadyl phosphate ultrathin nanosheets for high energy density and flexible pseudocapacitors. *Nature.Commun* **4,** 2431 (2013).
26. Yang, P. H. et al. Hydrogenated ZnO core-shell nanocables for flexible supercapacitors and self-powered systems. *ACS Nano* **7,** 2617 -2626 (2013).
27. Fang. J. et al., Metallic Few-Layered VS_2_ Ultrathin Nanosheets: High Two-Dimensional Conductivity for In-Plane Supercapacitors. *J. Am. Chem. Soc*. **133,** 17832–17838 (2011).

1. ^^ Correspondence and requests for materials should be addressed to K.I.O (email: [kozoemena@csir.co.za](mailto:kozoemena@csir.co.za)) [↑](#footnote-ref-2)
